# Weight Maintenance after Dietary Weight Loss: Systematic Review and Meta-Analysis on the Effectiveness of Behavioural Intensive Intervention

**DOI:** 10.3390/nu14061259

**Published:** 2022-03-16

**Authors:** Giovanna Flore, Antonio Preti, Mauro Giovanni Carta, Andrea Deledda, Michele Fosci, Antonio Egidio Nardi, Andrea Loviselli, Fernanda Velluzzi

**Affiliations:** 1 Obesity Unit, Department of Medical Sciences and Public Health, University of Cagliari, 09124 Cagliari, Italy; andredele@tiscali.it (A.D.); michele.fosci92@gmail.com (M.F.); alovise2@gmail.com (A.L.); fernandavelluzzi@gmail.com (F.V.); 2 Department of Neuroscience, University of Turin, 10124 Torino, Italy; antolink@yahoo.it; 3 Department of Medical Sciences and Public Health, University of Cagliari, 09124 Cagliari, Italy; maurogcarta@gmail.com; 4 Institute of Psychiatry, Universidade Federal do Rio de Janeiro, Rio de Janeiro 21941-901, Brazil; antonioenardi@gmail.com

**Keywords:** weight loss, weight loss maintenance, overweight, obesity, diet, low caloric diet, physical activity, exercise, behavioural approach, gender

## Abstract

After a low-calorie diet, only 25% of patients succeed in maintaining the result of weight loss for a long time. This systematic review and meta-analysis aims to explore whether patients undergoing intensive intervention during the maintenance phase have a greater preservation of the weight achieved during the previous slimming phase than controls. A bibliographic search was conducted using PubMed, Scopus, and Cochrane databases for clinical trials and randomised, controlled trials investigating the role of choice in weight-loss-maintenance strategies. Only studies with a follow-up of at least 12 months were considered. A total of eight studies, for a total of 1454 patients, was identified, each comparing a group that followed a more intensive protocol to a control group. Our metanalysis highlighted that an intensive approach even in the maintenance phase could be important to ensure greater success in the phase following the weight-loss period. However, it should be pointed out that the improvement was not so different from the trend of the respective controls, with a non-statistically significant mean difference of the effect size (0.087; 95% CI −0.016 to 0.190 *p* = 0.098). This finding, along with the observation of a weight regain in half of the selected studies, suggests this is a long work that has to be started within the weight-loss phase and reinforced during the maintenance phase. The problem of weight control in patients with obesity should be understood as a process of education to a healthy lifestyle and a balanced diet to be integrated in the context of a multidisciplinary approach.

## 1. Introduction

Obesity is a global problem with an impact on physical and mental health. It is associated with an increased risk of all-cause mortality and with morbidity for dyslipidaemia, type 2 diabetes, hypertension, coronary heart disease, stroke, liver and gallbladder disease, respiratory disease, sleep apnoea, osteoarthritis, and some cancers [1,2,3,4]; among the related complications of this condition, health costs [5,6], problems related to social relationship, and stigmatizing attitudes against people with obesity [7] are very important. Obesity represents a complex network of mutual influences among biological, psychosocial, and behavioural factors, which include genetic factors [8], gut microbiome [9,10], hypothyroidism [11], Cushing’s syndrome, polycystic ovary syndrome, sleep deprivation [12], neurological [13] and psychopathological [14] problems, as well as socioeconomic status and cultural influences [12]. Food and beverages choices and the consequent caloric intake are influenced by home environment, childcare, school, workplace, community, and urban environment, which could also impact the physical activity level and the caloric expenditure [15,16,17].

Clinical guidelines recommend that obesity-management programs consist of a low-calorie diet, increased physical activity, and behavioural style modification, according to an integrated multidisciplinary approach [18]. Nutritional intervention, including both caloric restriction and changes in eating habits consistent with a healthy model, such as the Mediterranean diet, is essential in weight reduction and in improving many obesity-related conditions [19,20]. However, in the context of weight-management interventions, combining physical exercise with dietary prescriptions results in better outcomes in terms of weight loss and other anthropometric or clinical changes than diet alone, establishing an important synergy between the two types of intervention [21].

It has been reported that, after a low-calorie diet to lose weight, only 25% of patients maintain this loss in the long term [22,23]. Greenway started from the assumption that this is a complex problem and that obesity could be considered a neurobiological disease with a psychological element. An obesogenic environment could impact cortico-limbic brain areas involved with learning and memory, pleasure and reward, mood, and emotion, favouring a compensatory food intake often accompanied by sedentary behaviours. In addition, personal motivation, confidence in personal ability and matching an ideal image, the value system, and expectations have been reported to influence weight maintenance. [22]. Factors that negatively influence weight loss and its maintenance include young age, smoking, higher BMI (body mass index), and low schooling [24,25]. Conversely, success in weight-loss maintenance has been associated with behavioural factors (high levels of physical activity, hypocaloric and hypolipemic diets, frequent weight monitoring), cognitive factors (reduced disinhibition, satisfaction with results, ability to self-regulate), personal traits (little novelty seeking), and long-term group help, such as face-to-face or web-based interaction [26,27,28,29].

The main strategies for maintaining a stable, long-term weight include high levels of physical activity, low-calorie, low-fat diet, regular breakfast, self-monitoring of weight, and maintaining a consistent eating pattern. Moreover, individuals with control over their environment and stimuli tend to have more sustained weight maintenance than those who perceive their lives to be regulated by something outside of their control [30]. In a systematic review, Ramage et al. highlighted the importance of combining three different strategies (reduced energy intake, physical activity, and behavioural therapy) in both the weight-loss and maintenance phases although behavioural therapy in the weight-maintenance phase represents a critical factor for long-term changes [27]. Older patients, whose primary motivation for weight loss is to improve health, are more compliant with ongoing care. However, social support and enjoyable activities requiring physical exercise may increase lifestyle changes and adherence to intervention programs aimed to maintain weight loss in motivated patients. In addition, body perception, increased self-confidence, and social support can increase self-motivation, favouring long-term results and improving quality of life [31,32].

The need for a support during the maintenance phase is demonstrated by the increasing development of applications that allow for self-monitoring, preferring systems that are beautifully designed and easy to use. Using self-monitoring, having a community, sharing on social media (i.e., offering social support), and using notifications (i.e., behavioural suggestions/indications) appear to be features associated with higher app quality. This shows that offering features to support specific technologies could improve the perceived functionality, aesthetics, and engagement of the app and lead to repeated use [26].

There are several studies on weight-maintenance strategies after weight loss [33], and a recent narrative review provided an overview of the most commonly used [34], such as (1) a low-caloric and low-fat diet [33] even if the macronutrients composition does not seem to influence the outcomes [35]; (2) physical activity, which, coupled with dietary intervention, gives better results than diet alone [36] but, unfortunately, does not find good long-term adherence [37]; (3) the use of drugs (orlistat, liraglutide, naltrexone/bupropion, and phentermine/topiramate), which can supplement lifestyle intervention in patients with a BMI > 30 kg/m^2^ or >27 kg/m^2^ with comorbidities [38]; and (4) bariatric surgery, which is currently considered the most effective therapy for long-term weight loss and for reducing comorbidities and mortality due to severe and complicated obesity but requires a careful nutritional surveillance to prevent and correct the frequent nutritional deficiencies [39]. Among other strategies, the following should be mentioned: acupuncture, acting on the mechanisms of appetite, if only in assisting other treatments [40]; continuous contact between the patient and health professionals, [41] and sharing with a group, including on social networks [41].

In the context of a multidisciplinary approach, behavioural and cognitive behavioural interventions are particularly important in helping patients to manage maladaptive eating or exercise behaviours, amplifying weight-loss results, but their role in the long term is still poorly understood [42].

This systematic review and meta-analysis was set up to investigate the effectiveness of weight-maintenance behavioural strategies tested in randomised, controlled trials. The population of interest consisted of patients who had undergone a successful dietary weight-loss treatment for overweight or obesity and were involved in a study on weight-maintenance intervention; the outcome of interest was the weight difference between cases and controls after weight-maintenance intervention at fixed follow-up (12 months).

## 2. Materials and Methods

### 2.1. Research Strategy

**Figure 1:**
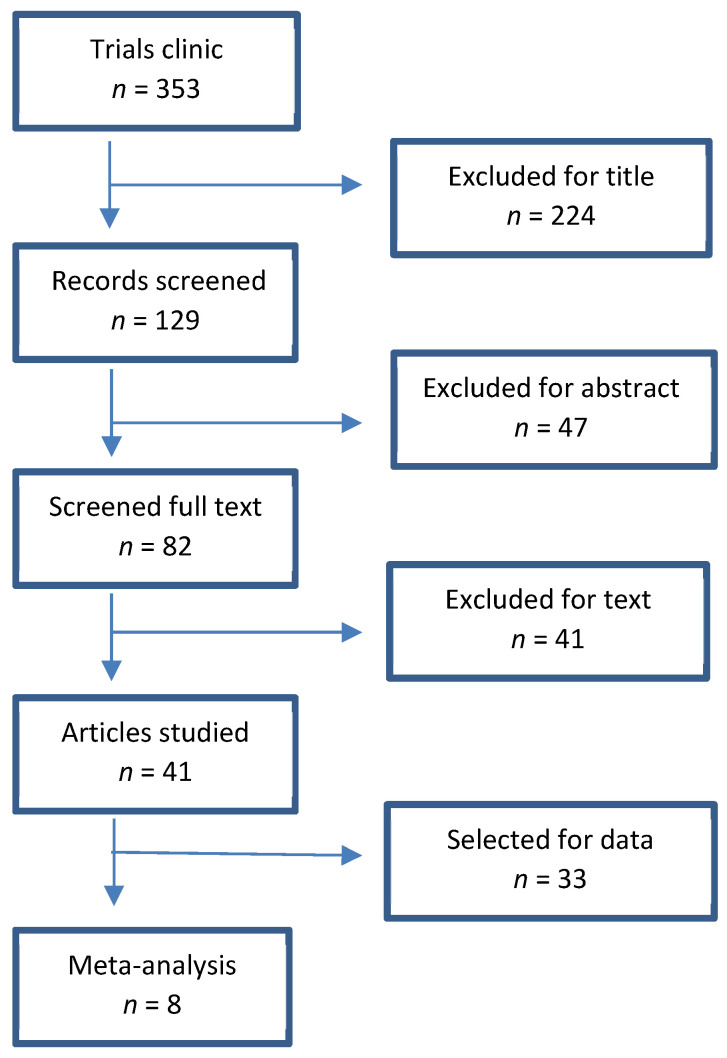
Flow diagram illustrating the process of our review, screening, and articles selection.

For this work, a bibliographic survey was performed in the search engines PubMed, Cochrane, and Scopus, supplemented by a manual, in-depth study of articles in the respective bibliographies and other supports (reviews on the topic). The survey was fulfilled to acquire randomised and controlled clinical trials published in the last 5 years (2016–2020), using the following keywords: weight loss, maintenance, exercise, physical activity. Studies were included when they dealt with individuals with an age greater than or equal to 18 years and without intellectual disabilities or psychiatric disorders due to increased complexity in treatment [43]. Moreover, we considered only studies with a follow-up of at least 1 year. A first screening was done based on title, the second screening after reading the abstract, and finally, the text of the selected studies was analysed. We considered only articles written in English (Figure 1).

Only the body weight value and its changes after treatment were taken as an outcome for the meta-analysis. For some data, the authors were contacted personally, and some of them supplemented their data with the body weight values, allowing us to standardize the measures.

### 2.2. Inclusion Criteria

Only randomised, controlled trials including adult people were considered in this study. All patients had previously undergone dietary treatment for weight reduction. Some studies included a first phase aimed at weight loss followed by a maintenance phase, while in other studies, patients who had lost weight within the previous year with other programmes were also enrolled for the maintenance program. No sex distinction was made; thus, some selected studies included only men or women, but most of them included both sexes. Finally, the selected studies used an intensive behavioural approach during the maintenance phase, with the aim of maintaining as much as possible the weight achieved during the previous phase of weight loss. The results were compared with those of a control group, which received a standard treatment. The data were sufficient and suitable to perform a meta-analysis.

### 2.3. Data Extraction and Quality Assessment

After selecting items that met the established criteria, we proceeded to data extraction and elaborated a table comprising the total number of patients included in each study, distinction by sex, and the body weight (mean value ± standard deviation) at recruitment and at the 12-month follow-up. In some studies, data were indicated as the weight value reached at the follow-up, while in other studies, data were indicated as the difference between the weight value at follow-up and at baseline. In the last case, we proceeded to contact the authors to have the weight values reached at the end of the study. Only two authors responded, allowing us to standardize the results to be included in the meta-analysis. Statistical examination was performed only on the part devoted to studies that brought a more intensive behavioural approach in following patients to identify whether their greater involvement was sufficient to increase the chances of success.

The effect measure of interest was the standardized mean difference between the active intervention groups and the control groups. Hedge’s g rather than Cohen’s d was used as the effect size for the analysis because it is unbiased. When possible, we calculated Hedge’s g from the means, standard deviations (SDs), and sample sizes presented in the article. When the standard error (SE) was reported in the study but not the SD, the latter was estimated using the following equation: SD = SE × $\sqrt{n}$, whereas, if the confidence interval was present, the SD was estimated using the following equation: $\sqrt{n}$ (upper Limit CI—lower Limit CI)/3.92 (at 95%).

Data were imported into the Comprehensive Meta-analysis program for quantitative synthesis. Because of the inevitable variability in the methods used in the different studies, a random analysis was performed. Funnel plot was used to inspect for indications of publication bias (asymmetry in the distribution of effect sizes). Egger’s test was not conducted because the articles were less than 10 and therefore less than those suggested by the recommendation’s asymmetry of Cochrane on testing for funnel plot.

## 3. Results

A total of 353 articles were found in the literature search, of which, after several screenings, namely the first for title and the second for abstract, 41 were eligible for text analysis. Among these, eight had eligibility criteria to be included in the meta-analysis (Figure 1). A contact via email with the authors of two of the eight articles (Nakata and Knauper) allowed us to obtain unpublished data and permitted their inclusion in the meta-analysis.

### 3.1. Characteristics of the Studies Included in the Meta-Analysis

The studies included in the meta-analysis enrolled a total of 1454 patients, of whom 990 were women (68%). Three studies were performed in the United States, two in Europe, one in Australia, one in Canada, and one in Japan. The mean age of patients ranged from 41 to 65 years.

In all studies, the protocol consisted in the comparison between two weight-maintenance programmes of different intensity, aimed at accompanying patients in the post- weight-loss phase through a hypocaloric diet. In particular, intervention groups received a treatment consisting of protocols of psychological and motivational intervention, nutritional support, and/or physical activity more intense than those commonly used for overweight-obesity and related pathologies, especially type 2 diabetes mellitus (Table 1).

The specific protocols used in the eight selected studies are described in detail below.

Williams’ treatment (2019) consisted of five consultations over the 12 months (four with a nutritionist and one with a sport physiologist) plus a paper support, while the controls received only paper materials [44]. Sniehotta’s protocol (2019) included a portal where patients daily entered their weight weekly, with both their physical activity measured by a pedometer and their food diary. They received with each data entry a text message commenting on it. A support phone call was allowed at the patient’s request. The controls received a SMS every 3 months with general suggestions [45]. Nakata (2019), after the weight-loss phase, performed a weight-maintenance phase, in which the control group did not receive any additional support, while the intervention group received Web support for daily monitoring of weight and physical activity. The system created a graph of the data and a report for evaluation and encouragement [46]. In the work of Dutton et al. (2017), after the phase of weight reduction (4 months), the maintenance phase included 12 intensive group sessions of 4 weeks, with counselling on issues related to diet, physical activity, maintenance strategies, and relapse prevention. The controls received only printed information material and the possibility of counselling by phone or email on request [47]. Crain’s protocol (2018) consisted of two phone calls in the first month and a book for protocol and data collection for the control group, while the intervention group received 10 biweekly calls, followed by 8 monthly calls, with no indication of nutrition and/or goals but encouragement of self-monitoring and physical activity [48]. Berk et al. (2018), after the period on a low-calorie diet, followed diabetic patients for weight maintenance with cognitive behavioural therapy (CBT), which aims to change dysfunctional thoughts with useful, lasting thoughts about self-image. Group sessions had a decreasing frequency over time. The controls instead followed the normal protocol for diabetic patients, with visits every 3–6 months [49]. Voils et al. (2018) subjected patients to a low-calorie diet and then followed them for 42 weeks, alternating group sessions every 4 weeks, with individual phone calls always every 4 weeks for psychological and practical support in the phase of weight maintenance. The control group did not receive any contact [50]. Finally, in the study of Knauper et al. (2020), all patients received GLB (group lifestyle balance), a treatment consisting of 12 weekly, one-hour group sessions for advice on nutrition, pedometer use, and ongoing support of diabetic patients. In the intensive treatment group, the if-then plan was added, which is a plan aimed at self-management of risk situations and choosing the strategies to adopt [51].

The table below shows the weight values at the beginning of the study (T0) and after one year of treatment (T12) both in intervention and in control groups of the eight studies (Table 2).

### 3.2. Weight Variations

**Figure 2:**
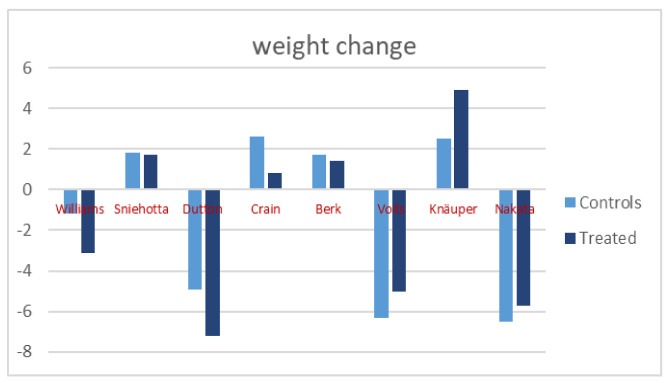
Weight changes between T0 and T12 in controls and treated patients in the eight studies.

As shown in Figure 2, both Williams and Dutton, during the maintenance phase, succeed in further reducing weight, with a significantly larger decrease in patients who had received more intensive treatment (−3.1 kg T/−1.2 kg C *p* = 0.02; −7.2 kg T/−4.9 kg C *p* = 0.02, respectively). In these protocols, the intensive treatment included sessions, often in groups, spread over the maintenance period in order to keep continuous contact with experienced health professionals who provided information and advice. In the study of Voils, there was weight loss in both groups, with a slightly greater effect in the controls (−5 kg T and −6.3 kg C). A similar result was observed in the study of Nakata, in which the further weight loss obtained in both groups was slightly higher in the control group (−5.7 kg T and −6.5 C), whose treatment consisted simply of daily self-monitoring of weight.

The protocols of Crain, Berk, Sniehotta, and Knauper were not able to maintain the weight loss, but patients followed more intensively had a reduced weight regain when compared to patients following normal protocols except in the study of Knauper. In Crain’s work (increase of 2.6 kg in controls and 0.8 in treated patients), patients received calls from the evaluation team frequently in the first months, then more and more rarely. In Berk’s work, in which the weight regain was very low (1.7 kg in controls and 1.4 kg in treated patients), there were frequent group sessions more frequently in the first months, then decreasing in frequency. A similar result was observed in Sniehotta’s study (increase of 1.8 kg in controls and 1.7 kg in treated patients), in which the health professionals asked for a daily self-monitoring of weight. Finally, all patients followed with Knauper’s protocol presented a weight gain, which was higher in patients receiving the if-then plan (4.9 kg in treated and 2.5 kg in controls) (Figure 2).

**Figure 3:**
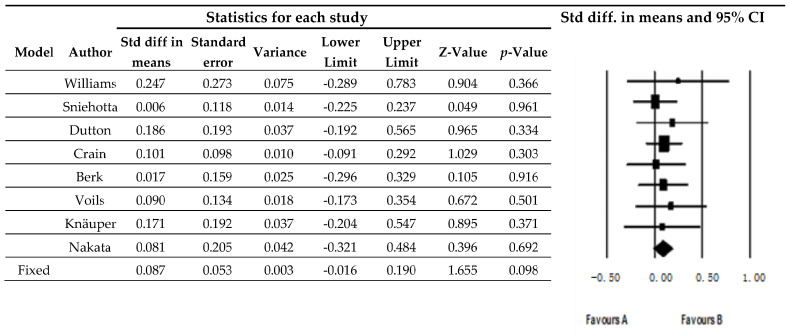
Meta-analysis of effectiveness of weight-maintenance strategies.

The meta-analysis of effect size yielded a mean difference of 0.087 (95% CI −0.016 to 0.190 *p* = 0.098), which is non-statistically significant (Figure 3).

Heterogeneity (differences between primary studies) was inexistent I^2^ = 0.

**Figure 4:**
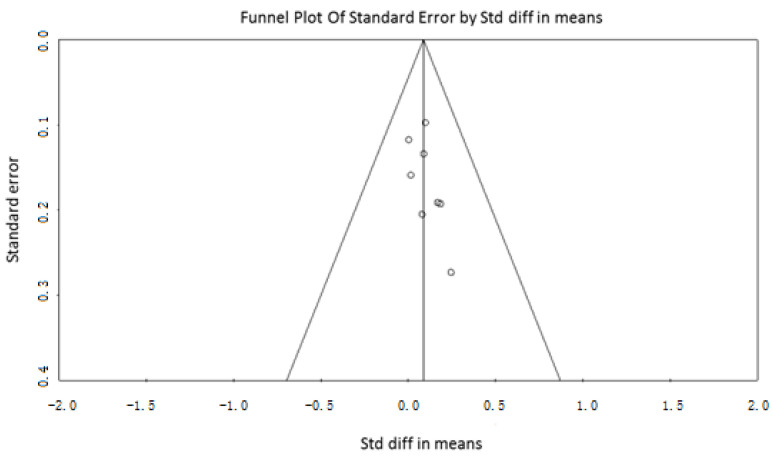
Funnel plots to ascertain evidence for publication bias.

Egger’s test was not conducted because the articles are less than 10 and therefore less than those suggested by the recommendations on testing for funnel plot asymmetry of Cochrane. The funnel plot (Figure 4) shows a good symmetry of the selected studies.

According to the Cochrane risk assessment tool of bias, the studies were adequate regarding the generation of randomization although some studies did not specify whether there was prediction in the assignment. In the different protocols, it was not possible to keep patients blind about the group to which they belonged; with regard to the operators, only in four studies did we ascertain adequate protection from detection bias where both the clinician who followed the patients and the researcher who analysed the data were unaware of the group to which the patient belonged. All outcomes were detected using appropriate methodology, and the groups presented a valid homogeneity. In six studies, there was an excellent adherence to the protocol; only in two studies was there a drop-out of more than 20%. Finally, six authors explicitly stated that they used an Intention to Treat analysis. (Table 3)

## 4. Discussion

The aim of this systematic review and meta-analysis is to establish the effectiveness of an intensive behavioural treatment in maintaining weight loss previously achieved in overweight and obese patients. To this purpose, we considered eight studies eligible for the meta-analysis, with a follow-up period of at least twelve months. Overall, some somewhat discordant results were obtained. In fact, although some of the selected studies found an improvement in intensively treated patients compared to the control group, the results of our meta-analysis did not reach statistical significance, suggesting that following patients even with an intensive approach in the maintenance phase does not seem sufficient to maintain the results previously obtained.

In addition to the motivation that drives people to lose weight and maintain the result over time, other factors should be considered. Among the many variables that could affect the results, there could be a different behavioural approach, a different need related to gender, and a different type of physical activity to be undertaken during the two phases of reduction and maintenance of the weight achieved.

### 4.1. Behavioural Approach

Some studies reported that following patients in the maintenance phase could be important to ensure a greater success in the post-weight-loss phase.

However, it should be emphasized that as regards the studies included in our metanalysis, the overall improvement observed in patients who received an intensive treatment during the maintenance phase did not significantly differ from the trend of respective controls.

In particular, four studies reported a further weight decrease in the maintenance phase, but only in two of them was it greater in intensively treated patients than in controls. A careful examination of these studies highlighted that the best results were obtained in the studies of Williams and Dutton, in which the intensive maintenance protocols were based on the continuous contact between the patients and the health professionals. During the sessions, often in groups, the patients received information and advice regarding a correct diet and lifestyle as well as on the prevention of relapses. On the other hand, the studies of Voils and Nakata did not show a better outcome in intensively treated patients than in control groups. In these two studies, the intensive protocols did not include a direct contact between the patients and the operators, but they were based on Web or phone contact.

These results might suggest that a different behavioural approach, more specifically a direct contact or not, could be important in influencing the outcome.

Regarding the other four selected studies, all showed the frequently observed weight regain that, however, in three studies, was lower though only slightly in patients treated with a greater involvement, often via the Web.

Among these studies, Crain’s study, in which the intervention group was followed with frequent phone calls encouraging self-monitoring and physical activity, showed the lowest weight regain in comparison with the control group. As for the other three studies, two of them, Sniehotta’s and Berk’s studies, which used a Web protocol and a group CBT protocol, respectively, showed a low weight regain similar in the two groups and only marginally lower in patients followed with direct contact in Berk’s study. Finally, in the study of Knauper, in which both groups were followed with behavioural sessions, while in the intensive protocol, a further behavioural support was added, the weight regain was even higher in more intensively treated patients than in controls. In this study, the authors concluded that the additional behavioural support provided to the intensive group was probably not strong enough to significantly improve the effect of the behavioural therapy per se. A similar conclusion was given by Berk et al., who attributed the lack of a greater effectiveness of CBT to the presence of usual care elements also somewhat helpful in the maintenance phase.

To summarize, behavioural intervention has been shown to be an effective therapeutical strategy in the weight-loss maintenance, but its effectiveness could have been influenced by the type and the intensity of the approach. Furthermore, on the basis of the not significant result of the meta-analysis and particularly of the observation of a weight regain in half of the selected studies, we hypothesized that the core of the problem is that the behavioural work should be started earlier in the phase of weight loss. From the first contact with the professional team, the patient should be provided with as much information as possible about diet and lifestyle, but especially, it is fundamental to make patients aware that obesity is a chronic and complex disease that needs a lifelong treatment. In this context, the behavioural intervention should be a long process, often leading to a radical change in lifestyle to be inserted in a multidisciplinary approach from the beginning and to be continued over time to consolidate the results.

Public health messages about weight management for long-term health, disease prevention, general well-being, or fitness improvement are ubiquitously endorsed. However, reasons such as improving appearance, conforming to external demands/needs (partner, doctor, peers), and avoiding social discrimination are also very common. Having a more positive body image, not feeling pressured or discriminated against, and losing weight primarily for autonomous and (internal reasons all could lead to a greater adherence to healthy behaviours and higher success rates [52].

On this issue, a behavioural intervention focussing on examining the nature of the goals and the quality of motivation behind the desire to lose weight can could prove useful at various points in the weight-management process in addition to considering the extent of a person’s motivational drive.

In weight control, as in other areas of behavioural medicine, only when individuals fully endorse behavioural goals and to the extent that such goals facilitate the satisfaction of autonomy, competence, and relationship needs, their efforts will most likely lead to effectively maintained behavioural change. Often, goals are set by health professionals or indirectly promoted by society but perhaps never fully internalized, leading to unsuccessful outcome [53].

### 4.2. Gender Difference

One aspect that should be considered when evaluating the effectiveness of behavioural intervention in the weight management is the gender difference.

There are not many studies in the literature evaluating the different approach in men and women when considering healthy and active lifestyle and specifically weight maintenance after weight loss.

Moreover, these studies are carried out more frequently on females. Out of 23 clinical trials, which included both sexes in the study, total female participation was more than double that of men, and this trend also regards studies that target physical activity.

Overall, these studies demonstrate a substantial difference in addressing the problem.

The higher retention rates of study participants indicate that it may be more difficult to attract men to join weight-loss programmes than women, but once they become involved, men will become seriously engaged in the program. Therefore, for men, it is important that programmes are attractive in order to promote the effectiveness of the interventions [54].

Men have a greater propensity to misperceive their weight; they are often satisfied with their physical condition even if they have overweight and sometimes obesity. As a result, they tend to underestimate their health risk and are less likely to consider weight-reduction treatment [55,56].

A study performed in the Mediterranean area found that although males showed a higher prevalence of overweight, females were more likely to be worried about body weight; however, this dissatisfaction seemed more related to body image and physical appearance than to health status [57].

Gender differences in weight-related attitudes and behaviours were also found in a U.S. work although they decreased as BMI increased from overweight to obesity values. In this study, men who attempted weight loss were 41% more likely than women to be successful, and they were more likely to exercise and eat less fat, while women were more likely to join a weight-loss programme, take prescription diet pills, follow a special diet, and eat more vegetables and fruits. This gender disparity deserves attention because men seem to benefit from weight loss, especially when undergoing a personalized program [58].

It is of note that a study performed on young Australian university students found that both men and women rate as more attractive a woman with less fat mass rather than an optimal ratio of lean-to-fat mass, associating this combination with optimal health; however on the other hand, for male bodies, the amount of muscle and fat mass chosen to optimize attractiveness was consistent with a physiologically healthy body composition [59].

Similar findings were reported in other studies. Poulimeneas [60] found that men drastically change their lifestyle by significantly increasing vigorous physical activity, while women do not drastically change their lifestyle but look for ways to modulate their routine.

Crane et al. [61] observed that both men and women consider weight loss a major need to improve health, but among women, a higher percentage reported the need to increase self-esteem.

Young et al. [62] studied the long-term behaviour of men given that they are the gender least likely to undertake weight-loss diets, which, in this protocol, lasted three months. Following a successful weight loss, after 3 years, almost the half of patients had a weight regain independently from having received or not a specific intervention for maintaining weight. This finding seems in contrast with the studies that report that men are inclined to change their lifestyle in the long term.

To summarize, clinical trials, despite a clear prevalence of females, show greater tendency of men to adhere to personalized protocols, especially when focused on physical activity, and in general to make significant lifestyle changes. Instead, women prefer to adhere to protocols, often group protocols, that include adjustments to their usual lifestyle. Nevertheless, in both sexes, the results seem not to be maintained in the long term, supporting the hypothesis that the work should be undertaken from the beginning in the phase of weight loss.

As for the studies included in our meta-analysis, they were in line with the data of the literature, having enrolled a prevalence of female patients. However, also when the study involved patients of both sexes, a different plan was not provided based on gender; nor were the results presented separately. Therefore, we are not able to identify any gender-related issue.

### 4.3. Physical Activity

A sedentary lifestyle is defined as fewer than 5000 steps per day, whereas an active lifestyle is understood to be when at least 8000–10,000 steps per day are achieved [21]. Frequent claims that exercise is more important for maintenance than for initial weight loss are not unanimously supported. Fundamentally, it is an area that is perhaps understudied. It would seem that habits formed during the weight-reduction phase and maintaining exercise above suggested levels to maintain health and/or lose weight may be determinants at this stage [63].

Adults and older adults should participate in ≥150 min/week of moderate to vigorous physical activity (MVPA) to prevent significant weight gain and reduce chronic disease risk; muscle-strengthening exercises are also recommended at least two days per week [64].

For patients seeking to maintain weight loss, high levels of physical activity (200–300 min/week of moderate intensity) were associated with better outcomes than lower levels (150 min/week). In addition, a greater weight maintenance was also associated with adherence to dietary plans, regular weighing, and engagement in support groups [65].

It should be considered that most studies are based on self-reported measurements, which often overestimate the physical activity level, while an objective assessment is essential to establish the optimal level needed for both weight loss and maintenance [66].

Unick et al. [67] reported that a weekly physical activity of 150–250 min, objectively assessed by means of an accelerometer, is sufficient to maintain the lost weight over time. Beyond this limit, there are no major differences.

Kerns et al. [68] observed 14 individuals who participated in the television programme *The Biggest Loser*. In this study, it was shown that people who had maintained discrete physical activity over time managed to maintain a good percentage of the weight lost during the competition against a comparable caloric intake, suggesting the main role in maintaining weight loss is played by physical activity.

Eaton et al. [69] showed that in patients treated with a lifestyle intervention for obesity, a physical activity of up to 126 min/week was still able to provide a greater weight maintenance than controls. Below this value, even if it was higher than that of controls, physical activity was not sufficient to ensure a greater weight maintenance.

Coleman et al. [70] subjected 51 patients, who had previously undergone bariatric surgery, to usual care or to a programme designed to achieve an ultimate MVPA goal of 150 min/week, with a series of exercises tailored to the specific needs of each patient and based on their abilities. All exercises had intensity/difficulty levels such that they could be performed safely and consistently at home. The results showed that intensively treated patients reached a significantly higher improvement in the objectively assessed fitness level, strength, and flexibility than controls, while weight remained virtually unchanged. The improvement also remained during the maintenance phase although when the monitoring became optional, patients of the intervention group reduced their activity level. The authors conclude that in order to improve the adherence, prescribing 3 to 4 days per week for 30 to 60 min of personalized exercises with a social support and monitoring component may be a more reasonable option than expecting that most post-bariatric patients are able to maintain the public health recommendations of 150 min per week of MVPA or 10,000 steps.

The Football Fans in Training (FFIT) programme, in the study of Gray et al. [71], engaged men in weight management through their interest in soccer and encouraged them in small increments of physical activity and dietary changes in daily life to support long-term weight-loss maintenance. The researchers concluded that indulging the patient’s interests and passions makes the related physical activity easy to perform and continue, thus facilitating the long-term outcomes.

Finally, regarding the studies selected for our meta-analysis, most of the intensive protocols included counselling sessions focused on physical activity and on the self-monitoring of physical activity level by means of different (mainly via Web) supports. Some studies used specific questionnaires, thus giving a self-reported measure. In other studies, patients were provided with pedometers or accelerometers, obtaining a more objective assessment of the amount of physical activity, in order to verify the achievement of the established goals. The results, presented as a secondary outcome in some studies, showed the lack of significant differences between intervention and control groups. Nevertheless, the study of Nakata found that patients who reported a greater increase in their objectively measured step count also lost more weight in the maintenance phase.

A calorie-restricted diet is associated with changes in certain chemical parameters, such as reductions in the amino acid tryptophan (TRP), a precursor of the neurotransmitter serotonin, and phenylalanine, a precursor of the tyrosine-dopamine pathway. Animal and human studies have shown that aerobic exercise can stimulate serotonin activity in the brain. Thus, by eating a low-carbohydrate meal, the amount of TRP entering the brain will be substantially less than normal, contributing to depression symptoms, such as mental fatigue and low mood. Moderate exercise could counteract the effect of a low-calorie and low-carbohydrate diet, increasing the production of neurotransmitters, such as serotonin, norepinephrine, adrenaline, and dopamine, thus contributing to mood elevation. However, only exercises practised occasionally as a recreational activity, with two- or three-day intervals, seem to exert this beneficial effect on general well-being, while intense training will negatively affect both mood and immune system function because it determines the production of ROS, which blocks the activity of vit B4 and interrupts the synthesis of TRP. Therefore, it is necessary to achieve a balance between dietary habits and the optimal dose of physical activity [72].

Appetite and appetite-related hormonal changes after exercise are also important both in men and women. Changes in perceptions of appetite, energy intake, and macronutrient composition in response to acute and chronic exercise stimuli are not modulated by body adiposity levels or sex; individuals with higher levels of habitual physical activity may show better sensitivity of the appetite-control system through better compensatory adjustments for energy content and food density. However, there are multiple discrepancies in appetite-related hormonal outcomes, making it difficult to summarize how adiposity, sex, and habitual physical activity modulate exercise-induced changes in these hormones. [73].

To summarize, the results of the reviewed studies indicate that in the weight-maintenance phase, it is advisable to achieve a physical activity (moderate to vigorous) level of at least 150 min/week, better if distributed in 3–5 weekly sessions of 30–60 min, while a course exceeding 250 min/week may not be necessary. It is fundamental to adapt exercises to patient’s abilities and passions in order to obtain a greater adherence and probability of maintaining the behaviour in the long term. Therefore, it would be appropriate to carefully study patients’ abilities when assigning the exercises and even better to take advantage of the attractions for the patient and propose alternatives that support, for example, the passion for a particular sport or team. In fact, it can be inferred that a constant physical activity can be decisive in the weight-maintenance phase or, at least, delegate to it the main role in this phase since the strict control of energy intake is no longer fundamental.

We are aware that our study has some limitations. First and foremost is the paucity of the studies included in the meta-analysis. This is mainly due to our choice to limit the research only to the last 5 years since we felt it important to focus on the most recent behavioural approaches.

Another impactful screening was the choice of a minimum 12-month follow-up. We found many studies that had 3 to 6 months as their experimental time, a period that we assessed as too short to demonstrate weight consolidation.

In addition, the review was limited to the scientific literature written in English due to time and resource constraints for translation from other languages. As with any systematic review, the inclusion and exclusion are based on the reviewer’s decision.

## 5. Conclusions

The aim of this study is to evaluate whether following a patient affected by overweight or obesity in an intense way after having lost weight could lead to a greater maintenance of the previously obtained weight loss.

Despite the better outcome reported in some studies included in the meta-analysis, the overall results show the lack of significant differences between the intervention and the control groups.

This finding, along with the observation of a weight regain in half of the selected studies, suggests that this is a very complex issue and that several factors could influence the outcome.

Among these factors, a behavioural approach seems to play a key role.

Starting the work focused on motivation and awareness of the importance of a healthy lifestyle on both physical and psychological health from the first contact between the patients and the health professionals could improve the long-term results.

With regard to the methodology, our analysis highlighted that face-to-face contact gives the best results although technology support could be useful in the monitoring of some specific issues, such as physical activity level.

In addition, the analysis of the literature data showed the importance of tailoring the protocols on the basis of sex-specific needs and preferences. Furthermore, a regular physical activity performed according to the international guidelines, while also finding a pleasant and gratifying typology, seems to be fundamental to guarantee adherence in the long term [74,75].

Further long-term studies designed with protocols based on a multidisciplinary approach that takes account of these factors are needed for a better understanding of this crucial health problem.

## Figures and Tables

**Table 1:** Weight maintenance protocols used in the eight studies.

Author	Country	Year	Patients (Females)	Previous Weight Loss	Intervention Group Protocol	Control Group Protocol
Williams	Australia	2019	54 (54)	**	Nutritional and sport consultations plus a paper support	Paper material
Sniehotta	England (Europe)	2019	288 (223)	**	Portal where patients entered weight, physical activity, and food diary followed by a comment	SMS every 3 months
Dutton	USA	2017	108 (103)	*	Group counselling sessions on diet, physical activity, and strategies to manage weight maintenance	Printed material and phone/mail counselling on request
Crain	USA	2018	419 (342)	**	More frequent phone calls encouraging for physical activity monitoring	Less frequent phone calls plus a book
Berk	Holland (Europe)	2018	158 (88)	*	Group sessions of cognitive behaviour therapy with decreasing frequency	Usual care for diabetes
Voils	USA	2018	222 (34)	*	Group sessions alternated with phone calls for psychological and practical support	No support
Knauper	Canada	2020	110 (87)	*	Group sessions for nutritional and physical activity support plus an if-then plan to manage at-risk situations	No if-then plan
Nakata	Japan	2019	95 (59)	*	Daily monitoring of weight and physical activity via the Web	No support

* Weight-loss phase included in the study; ** weight loss obtained with other programmes.

**Table 2:** Randomised trials of maintenance interventions included in the meta-analysis.

Author	Number Patients			Age (Mean ± SD)		Weight of Treated (Mean ± SD)		Weight of Controls (Mean ± SD)	
	Total	Treated	Controls	Treated	Controls	T0	T12	T0	T12
Williams	54	28	26	47.3 ± 1.8	47.3 ± 1.8	68.7 ± 8.9	65.6 ± 8.5	68.6 ± 6.7	67.4 ± 6.7
Sniehotta	288	144	144	42.0 ± 11.6	41.6 ± 11.4	85.1 ± 17.5	86.8 ± 18.2	85.2 ± 15.7	87 ± 16.7
Dutton	108	52	56	52.13 ± 11.75	51.18 ± 14.22	92.4 ± 13.8	85.2 ± 13.2	92.7 ± 12.9	87.8 ± 11.7
Crain	419	209	210	46.6 ± 10.9	46.3 ± 10.3	80.1 ± 15.8	80.9 ± 16.7	79.4 ± 16.6	82 ± 17.9
Berk	158	83	75	52.3 ± 11.3	55.2 ± 9.3	96.3 ± 19.3	97.7 ± 20	96.8 ± 22.5	98.5 ± 15.3
Voils	222	110	112	61.5 ± 8.3	62.0 ± 8.3	109.3 ± 19.5	104.3 ± 13.9	112.2 ± 21.7	105.9 ± 14.8
Knäuper	110	51	59	50.22 ± 11.97	50.22 ± 11.97	80.8 ± 12.7	85.7 ± 14.4	85.8 ± 12.9	88.3 ± 13.4
Nakata	95	47	48	54.7 ± 6.6	57.0 ± 5.7	74.7 ± 10.6	69 ± 10.9	74.2 ± 8.1	67.7 ± 8.7

**Table 3:** Risk assessment Tool of Bias.

Study Quality-Assessment Tools															
Author	Year	1	2	3	4	5	6	7	8	9	10	11	12	13	14
Williams	2019	YES	YES	YES	YES	YES	NO	YES	NS	NS	YES	YES	YES	YES	YES
Sniehotta	2019	YES	YES	YES	YES	YES	YES	YES	YES	YES	NS	YES	NO	YES	YES
Dutton	2017	YES	YES	YES	NO	NO	YES	YES	YES	YES	YES	YES	YES	YES	YES
Crain	2018	YES	YES	NO	NO	NO	YES	YES	NS	YES	YES	YES	NO	YES	NS
Berk	2018	YES	NS	NS	YES	YES	YES	NO	NO	YES	YES	YES	YES	YES	YES
Voils	2018	YES	YES	NS	NO	NS	YES	YES	NS	NS	YES	YES	YES	YES	NS
Knäuper	2020	YES	YES	NS	NO	YES	NS	NO	YES	NS	YES	YES	NS	NS	NS
Nakata	2019	YES	YES	YES	NO	NS	YES	YES	NS	NS	YES	YES	NS	YES	YES

1 Was the study described as randomised, a randomised trial, a randomised clinical trial or an RCT?; 2 Was the method of randomization adequate (i.e., use of randomly generated assignment)?; 3 Was the treatment allocation concealed (so that assignments could not be predicted)?; 4 Were study participants and providers blinded to treatment group assignment?; 5 Were the people assessing the outcomes blinded to the participants’ group assignment?; 6 Were the groups similar at baseline on important characteristics that could affect outcomes (e.g., demographics, risk factors, comorbid conditions)?; 7 Was the overall drop-out rate from the study at endpoint 20% or lower of the number allocated to treatment?; 8 Was the differential drop-out rate (between treatment groups) at endpoint 15% or lower?; 9 Was there high adherence to the intervention protocols for each treatment group?; 10 Were other interventions avoided or similar in the groups (e.g., similar background treatments)?; 11 Were outcomes assessed using valid and reliable measures, implemented consistently across all study participants?; 12 Did the authors report that the sample size was sufficiently large to be able to detect a difference in the main outcome between group with at least 80% power?; 13 Were outcomes reported or subgroups analysed prespecified (i.e., identified before analyses were conducted)?; 14 Were all randomised participants analysed in the group to which they were originally assigned (i.e., did they use an intention-to-treat analysis)?; Possible answers: YES, NO, NS (not specified).

## Data Availability

Not applicable.
